# Prediction of New-Onset Atrial Fibrillation in Patients with Acute Myocardial Infarction: A Machine Learning Approach

**DOI:** 10.3390/medsci14060626

**Published:** 2026-09-30

**Authors:** Nadejda Chiriliuc, Alexandru Corlateanu, Oleg Arnaut, Ion Esanu, Lilia David, Daniela Bursacovschi

**Affiliations:** 1 Department of Cardiac Surgery Intensive Care, Public Healthcare Institution—Institute of Cardiology, MD-2025 Chisinau, Moldova; nadeachiriliuc@gmail.com (N.C.); ionesanu10@gmail.com (I.E.); 2 Department of Respiratory Medicine and Allergology, Nicolae Testemitanu State University of Medicine and Pharmacy, MD-2004 Chisinau, Moldova; 3 Department of Human Physiology and Biophysics, Nicolae Testemitanu State University of Medicine and Pharmacy, MD-2004 Chisinau, Moldova; oleg.arnaut@usmf.md; 4 Bioinformatics and Computational Medicine Laboratory, Nicolae Testemitanu State University of Medicine and Pharmacy, MD-2004 Chisinau, Moldova; 5 National Cancer Registry, Public Healthcare Institution—Oncological Institute, MD-2025 Chisinau, Moldova; 6 Laboratory of Cardiac Arrhythmias and Cardiac Emergencies, Public Healthcare Institution—Institute of Cardiology, MD-2025 Chisinau, Moldova; likadav27@yahoo.com; 7 Department of Heart Failure, Public Healthcare Institution—Institute of Cardiology, MD-2025 Chisinau, Moldova; daniela.bursacovschi@gmail.com

**Keywords:** atrial fibrillation, acute myocardial infarction, machine learning algorithms

## Abstract

**Background/Objectives**: New-onset atrial fibrillation is a common complication of acute myocardial infarction and is associated with increased mortality, heart failure, and recurrent cardiovascular events. Prediction models for the occurrence of atrial fibrillation may facilitate early risk stratification, improve patient monitoring, and support personalized therapeutic decision-making in clinical practice. **Methods**: A prospective study included 150 patients with acute myocardial infarction admitted within 24 h of symptom onset. Clinical, laboratory, electrocardiographic, and echocardiographic data were collected, and a machine learning model was developed to predict new-onset atrial fibrillation. Model performance was evaluated using ROC-AUC, while SHAP analysis was applied to identify and quantify the contribution of individual predictors. **Results**: The machine learning model demonstrated excellent predictive performance, achieving an accuracy of 97.0%, ROC-AUC of 0.991, and precision–recall AUC of 0.991 in the independent test set. SHapley Additive Explanations (SHAP) and permutation importance analyses identified the E/e’ ratio, left ventricular mass index, left ventricular ejection fraction, left atrial volume index, oxidative stress markers (malondialdehyde and superoxide dismutase), NT-proBNP, and complete revascularization as the most influential predictors of new-onset atrial fibrillation after acute myocardial infarction. **Conclusions**: Machine learning combined with SHAP-based explainability showed high potential for predicting new-onset atrial fibrillation after acute myocardial infarction. This approach may improve individualized risk assessment and clinical decision-making, pending validation in larger multicenter cohorts.

## 1. Introduction

Several studies have reported a significant increase in the incidence of atrial fibrillation (AF) during acute myocardial infarction (AMI). However, it remains unclear whether newly onset AF carries the same implications for short-term clinical outcomes as well as long-term prognosis. A precise understanding of this phenomenon is essential for appropriate risk stratification and for optimizing therapeutic strategies, including decisions regarding anticoagulation and overall patient management [1,2]. In 2021, atrial fibrillation and atrial flutter contributed substantially to the global burden of disease, with a total of 4,484,926 new cases reported worldwide. The age-standardized incidence rate was 52.12 per 100,000 individuals. In addition, the overall prevalence reached 52,552,045 cases, with an age-standardized prevalence rate of 620.51 per 100,000 [3]. AF is associated with a markedly increased risk of adverse cardiovascular events, including AMI [4]. Prospective cohort studies have shown that individuals with AF have a higher likelihood of developing AMI compared with those without this arrhythmia. Furthermore, multiple investigations have demonstrated an increased occurrence of AF both in the immediate period following myocardial infarction and during long-term follow-up [5]. Reported rates of de novo AF in the setting of acute coronary syndrome vary widely, ranging from 2.3% to 21% [6]. New-onset symptomatic AF is commonly observed within the first few days following AMI; however, silent forms appear to be approximately three times more prevalent. In a study conducted by Stamboul et al., which employed continuous ECG monitoring for at least 48 h after admission for myocardial infarction, 135 out of 849 patients (16%) developed silent AF, compared with only 45 out of 849 (5%) who presented with symptomatic AF [7]. Patients with silent AF were significantly older, more frequently female, and less likely to be smokers. Similarly to symptomatic AF, the silent form was associated with an unfavorable prognosis at one year after acute coronary syndrome, with a marked increase in cardiovascular mortality and hospitalizations for heart failure compared with patients without AF (5.7% vs. 2.0%, *p* < 0.001 and 6.6% vs. 1.3%, *p* < 0.001). Wi et al. further defined transient new-onset AF as arrhythmia occurring in the setting of myocardial infarction, without prior history and with no documentation one month after hospital discharge (observed in 4.8% of patients). This transient form of AF was associated with the poorest clinical outcomes, serving as a strong independent predictor of major adverse cardiovascular events and mortality in patients with myocardial infarction at one month, as well as at two- and five-year follow-up [8].

The development of predictive models represents a promising strategy for risk stratification in patients with AMI. By integrating clinical, electrocardiographic, echocardiographic, and laboratory parameters, predictive models can identify vulnerable patients during the early stages of hospitalization, allowing clinicians to implement closer monitoring and individualized preventive interventions. Wang et al. demonstrated that electrocardiographic markers, particularly P-wave amplitude in lead V1, can be incorporated into a predictive model with good discriminative ability for new-onset AF after AMI [9]. Furthermore, recent investigations have focused on constructing nomograms and multivariable prediction tools to estimate the risk of AF development after percutaneous coronary intervention in patients with AMI. Zhang et al. developed and validated a nomogram that showed satisfactory predictive performance and may support clinical decision-making in routine practice [10]. The growing application of artificial intelligence and machine learning techniques in cardiology has further enhanced the potential of predictive modeling. Machine learning algorithms can analyze complex nonlinear relationships among numerous clinical variables and identify hidden patterns associated with adverse cardiovascular events. Jeong et al. demonstrated that dynamic machine learning models can accurately predict clinical outcomes after acute myocardial infarction, highlighting the potential of these approaches for improving personalized patient management [11].

However, most existing models were constructed using traditional statistical approaches and are characterized by several important limitations. Conventional scores generally assume linear relationships between predictors and outcomes, which may fail to capture the complex interactions among clinical, laboratory, echocardiographic, and electrocardiographic variables involved in the development of new-onset atrial fibrillation. Also, many currently available models were derived from relatively small cohorts and often demonstrate limited generalizability when applied to external populations. Recent advances in artificial intelligence have demonstrated superior predictive performance compared with traditional statistical risk scores in various cardiovascular conditions [12]. However, one of the main challenges limiting the clinical implementation of artificial intelligence models is their lack of interpretability. To address this issue, explainable artificial intelligence techniques, particularly SHapley Additive Explanations (SHAP), have been increasingly integrated into predictive models. SHAP enables the identification and quantification of the contribution of each variable to the final prediction, thereby improving transparency and clinician confidence in model outputs [13]. SHAP-based approaches have been applied to arrhythmia prediction and atrial fibrillation risk assessment, prediction of heart failure, cardiotoxicity, etc., providing insights into the relative importance of electrocardiographic, laboratory, and echocardiographic variables [14,15]. Given the limited explainability of existing prediction models for new-onset atrial fibrillation after acute myocardial infarction, it would be valuable to investigate whether SHAP analysis can improve understanding of the factors driving atrial fibrillation development in this population. Such an approach could not only enhance predictive accuracy but also provide clinically meaningful explanations that support individualized risk stratification and decision-making.

## 2. Materials and Methods

This prospective investigation was carried out at the Institute of Cardiology in Chișinău, Republic of Moldova. Ethical approval was obtained from the Ethics Committee of the Nicolae Testemițanu State University of Medicine and Pharmacy (session dated 12 April 2018, approval No. 54). Eligibility was restricted to patients presenting within 24 h from symptom onset. The research protocol adhered strictly to the ethical standards outlined in the Declaration of Helsinki. Individuals were excluded if they had a non-cardiac condition limiting life expectancy to under two years, a history of AF, cognitive impairment, substance abuse or refusal to participate in the study. During the study enrolment period from April 2018 to December 2018, 232 patients admitted with acute myocardial infarction were screened. Of these, 82 patients were excluded based on the predefined exclusion criteria mentioned above. The remaining consecutive patients (aged 18 years and older) constituted the final study population and were divided into two groups: 75 consecutive patients who developed new-onset atrial fibrillation with a maximum in-hospital follow-up of 7 days (Group I) and 75 consecutive patients who remained in sinus rhythm (Group II).

All subjects underwent comprehensive clinical assessment, laboratory testing, and echocardiographic evaluation. To ensure the temporal relationship between predictors and outcome, all clinical, laboratory, echocardiographic, electrocardiographic, angiographic, and treatment-related variables included in the predictive model were collected at baseline, during the initial hospital admission for acute myocardial infarction, before the occurrence of new-onset atrial fibrillation. The echocardiographic examination was conducted by the same cardiologist using a General Electric Vivid E95 ultrasound machine (GE VINGMED ULTRASOUND A/S, Horten, Norway). The prediction time point was defined as the time of initial clinical evaluation after hospital admission and before any documented episode of new-onset atrial fibrillation.

Laboratory parameters, including NT-proBNP, were obtained at the time of initial admission before the development of new-onset atrial fibrillation. Echocardiographic assessment was performed during the index hospitalization before the diagnosis of new-onset atrial fibrillation, according to routine clinical practice. Angiographic characteristics and revascularization-related variables were recorded from the index coronary angiography and PCI procedure performed during the acute MI admission. Treatment variables, including beta-blocker and SGLT2 inhibitor use, were defined based on therapy initiated before or during the early management phase of the index hospitalization and documented prior to new-onset atrial fibrillation occurrence. Enrollment was performed using a random allocation process.

### Statistical Analysis

Data processing and statistical evaluation were performed using RStudio (version 2024.09.1+394) alongside Python (version 3.12.3), enabling a fully reproducible analytical workflow. Continuous variables were described using appropriate summary measures such as mean, standard deviation, median, interquartile rangeand range.

The prediction time point was defined as admission to the intensive care unit. All predictor variables were restricted to information available before or at this time point. Variables recorded after intensive care unit admission were not included in the prediction model. To identify potential predictors, variables related to cardiovascular risk profile, serological and oxidative stress biomarkers, echocardiographic parameters, previous cardiovascular medication use, treatment dosage, and clinical and angiographic features of acute myocardial infarction were evaluated. The model-development workflow used 32 predefined clinically relevant predictors. The full dataset included 150 participants, with equal numbers of patients with new-onset atrial fibrillation and sinus rhythm. For model development and evaluation, the cohort was divided into a 120-patient development set and a 30-patient independent internal hold-out test set using stratified random sampling. Thus, the final test evaluation was performed only on the 30 patients held out from model development. Within the development set, 11 candidate classifiers were compared using repeated stratified 5-fold cross-validation repeated 5 times. Extra Trees achieved the numerically highest mean balanced accuracy (0.947 ± 0.044), followed by Linear SVC (0.935 ± 0.053), logistic regression (0.932 ± 0.046), RBF SVC (0.920 ± 0.045), and histogram-based gradient boosting (0.918 ± 0.067). ROC-AUC values were similarly high for the leading models: Extra Trees 0.992 ± 0.010, RBF SVC 0.990 ± 0.015, Linear SVC 0.989 ± 0.025 and logistic regression 0.987 ± 0.021. Paired comparison of validation-fold balanced accuracy showed no statistically significant difference between logistic regression and Extra Trees (*p* = 0.084), Linear SVC (*p* = 0.848), RBF SVC (*p* = 0.169), histogram-based gradient boosting (*p* = 0.285) or gradient boosting (*p* = 0.113). Logistic regression was therefore retained as the final model because it provided high discrimination while preserving a simpler and more interpretable model structure suitable for a small clinical dataset.

The final logistic-regression model was then fitted on the 120-patient development set and evaluated once on the independent internal test dataset comprising 30 patients. The test set contained 15 patients with sinus rhythm and 15 patients with atrial fibrillation. The confusion matrix demonstrated strong discriminative performance. Among the 15 patients with sinus rhythm, 14 were correctly classified and one was incorrectly predicted as having atrial fibrillation. All 15 patients with atrial fibrillation were correctly identified, with no false-negative classifications. Overall accuracy and balanced accuracy were both 97.0%, the F1-score for atrial fibrillation was 0.968 and ROC-AUC was 0.991.

The predictive model developed for identifying the risk of new-onset atrial fibrillation in patients with acute myocardial infarction was assessed using standard discrimination metrics, including the area under the receiver operating characteristic curve (AUC-ROC). To enhance model interpretability, SHAP was applied to quantify the influence of individual variables on the prediction outcome. Global and local SHAP analyses were performed to determine the most relevant predictors associated with atrial fibrillation occurrence and to evaluate their contribution across different risk profiles. Graphical representations, such as SHAP summary plots and dependence plots, were used to visualize feature importance and explore potential interactions between clinical, laboratory, electrocardiographic and echocardiographic parameters.

The final model retained for testing was logistic regression with class weighting, selected after comparison with LDA, QDA, Gaussian naive Bayes, k-nearest neighbors, Linear SVC, RBF SVC, decision tree, Extra Trees, gradient boosting and histogram-based gradient boosting. Preprocessing was performed inside scikit-learn pipelines using median imputation and standard scaling for numerical predictors and most-frequent imputation with categorical encoding for categorical variables. Hyperparameters were not extensively tuned because of the small sample size; instead, predefined/default model settings were compared to reduce overfitting risk.

## 3. Results

### 3.1. General Characteristics of the Study Population

The study population consisted of 150 patients diagnosed with acute myocardial infarction. Men represented the majority of the cohort, accounting for 100 participants (66.7%, 95% CI: 59–74), while women comprised 50 participants (33.3%, 95% CI: 26–41). In terms of place of residence, 72 individuals (48.0%, 95% CI: 40–56) lived in urban areas and 78 (52.0%, 95% CI: 44–60) resided in rural settings. Participants ranged in age from 37 to 90 years, with a median age of 67.0 years (IQR = 13.0). Stratification by age revealed that approximately 58 patients (38.7%) were younger than 65 years, 72 patients (48.0%) were between 65 and 79 years of age, and 20 patients (13.3%) were aged 80 years or older. Assessment of lifestyle characteristics indicated that low levels of daily physical activity predominated within the cohort. A sedentary or minimally active lifestyle was reported by 107 participants (71.3%), whereas moderate physical activity was observed in 41 individuals (27.3%). Body weight measurements varied between 55 and 132 kg, with a median value of 86.5 kg (IQR = 22.8). The median body mass index was 29.0 kg/m^2^ (IQR = 8.0), ranging from 18.8 to 44.0 kg/m^2^. Obesity, defined as a BMI exceeding 30 kg/m^2^, was present in 65 patients (43.3%). When participants were classified according to nutritional status, overweight emerged as the most common category, affecting 55 individuals (36.7%). Both normal weight and class I obesity were observed in 36 participants each (24.0%). Furthermore, an enlarged waist circumference, a marker of central adiposity associated with increased cardiometabolic risk, was identified in 69 patients (46.0%). A positive cardiovascular family history was documented in 16 participants (10.7%). Evaluation of coexisting medical conditions demonstrated that arterial hypertension was the most prevalent comorbidity, affecting 102 patients (68.0%). Type 2 diabetes mellitus was recorded in 37 individuals (24.7%), reflecting the substantial burden of metabolic disorders among patients with acute myocardial infarction. Previous ischemic stroke was reported by 15 patients (10.0%), while peripheral arterial disease was identified in seven cases (4.7%). Regarding prior cardiovascular events, a history of myocardial infarction was noted in 15 patients (10.0%), and chronic heart failure was present in 42 patients (28.0%). These findings underscore the high prevalence of cardiovascular comorbidities and risk factors within the study cohort.

A detailed overview of the cardiovascular risk factors and comorbid conditions identified in the study population is provided in Table 1.

Within the overall study population, ST-segment elevation myocardial infarction represented the most common clinical presentation, being observed in 104 patients (69.3%), whereas non-ST-segment elevation myocardial infarction was identified in 46 patients (30.7%). Based on the etiological classification, type 1 myocardial infarction predominated, accounting for 147 cases (98.0%). Periprocedural myocardial infarctions were uncommon, with one patient (0.7%) classified as type 4 MI and two patients (1.3%) as type 5 AMI. Cardiogenic shock was documented in 10 patients (6.7%).

Analysis of infarct localization demonstrated that anterior wall involvement was the most frequently encountered pattern, affecting 74 patients (49.3%). Inferior wall infarction was recorded in 61 patients (40.7%), while apical involvement was less common and was observed in 15 cases (10.0%). Consistent with these findings, the left anterior descending artery was the vessel most often identified as responsible for the acute event, being implicated in 65 patients (43.3%). The right coronary artery was the culprit vessel in 53 cases (35.3%), whereas the left circumflex artery was involved in 27 patients (18.0%). Left main coronary artery disease was infrequent and was detected in only five patients (3.3%). Multivessel coronary artery disease was present in 125 patients (83.3%), indicating a high burden of coronary atherosclerosis across the cohort.

The median SYNTAX score was 28.0 (IQR = 12.0), with values ranging from 14.0 to 44.0. According to SYNTAX score categories, 37 patients (24.7%) were classified as having low anatomical complexity, 56 patients (37.3%) had intermediate complexity, and 57 patients (38.0%) were assigned to the high-complexity category. Overall, intermediate and high SYNTAX scores were identified in the majority of patients.

Regarding reperfusion therapy, 89 patients (59.3%) underwent revascularization during the initial angiographic procedure. Complete revascularization was achieved in 48 patients (32.0%). Concerning treatment delay, 13 patients (8.7%) were revascularized within the first 3 h after symptom onset, a further 13 patients (8.7%) underwent intervention between 3 and 6 h, and 22 patients (14.7%) received revascularization after more than 6 h. Delayed presentation was reported in 102 patients (68.0%).

At the time of admission, several classes of cardiovascular medications were already being used by study participants. ACE inhibitors or angiotensin receptor blockers were prescribed to 69 patients (46.0%), beta-blockers to 46 patients (30.7%), and combined beta-blocker plus ACE inhibitor/ARB therapy to 29 patients (19.3%). Statin treatment was recorded in 46 patients (30.7%), while diuretics were administered to 33 patients (22.0%). SGLT2 inhibitors and antiplatelet agents were used by 34 (22.7%) and 32 patients (21.3%), respectively. Other medications, including calcium channel blockers and gastroprotective agents, were reported in 29 (19.3%) and 33 patients (22.0%), respectively.

In the analysis of baseline characteristics and comorbidities, statistically significant differences between the two study groups were observed for peripheral artery disease, hypertension grade, anemia, and dyslipidemia (Table 2). Peripheral artery disease was more frequent in Group I (with newly onset AF) than in Group II or patients with sinus rhythm (9.3% vs. 0%; *p* = 0.014). The distribution of hypertension severity differed significantly between groups (*p* = 0.004), with grade III hypertension being more frequent in Group I (40.0% vs. 22.7%), whereas grade II hypertension predominated in Group II (48.0% vs. 21.3%). Anemia was substantially more prevalent in Group I (32.0% vs. 5.3%; *p* < 0.001), while dyslipidemia was more frequent in Group II (94.7% vs. 70.7%; *p* < 0.001).

### 3.2. Development of an Artificial Intelligence Model for Predicting New-Onset Atrial Fibrillation Following Acute Myocardial Infarction

To identify potential predictors, variables related to the cardiovascular profile and cardiovascular risk factors were evaluated alongside serological and echocardiographic parameters, previous cardiovascular medication use and treatment dosage, and clinical and angiographic features of acute myocardial infarction, including the number of diseased coronary vessels, the culprit artery, the extent of coronary artery involvement, and the location of myocardial injury. The prediction model was developed using data from 150 participants, with no missing data recorded.

**Figure 1:**
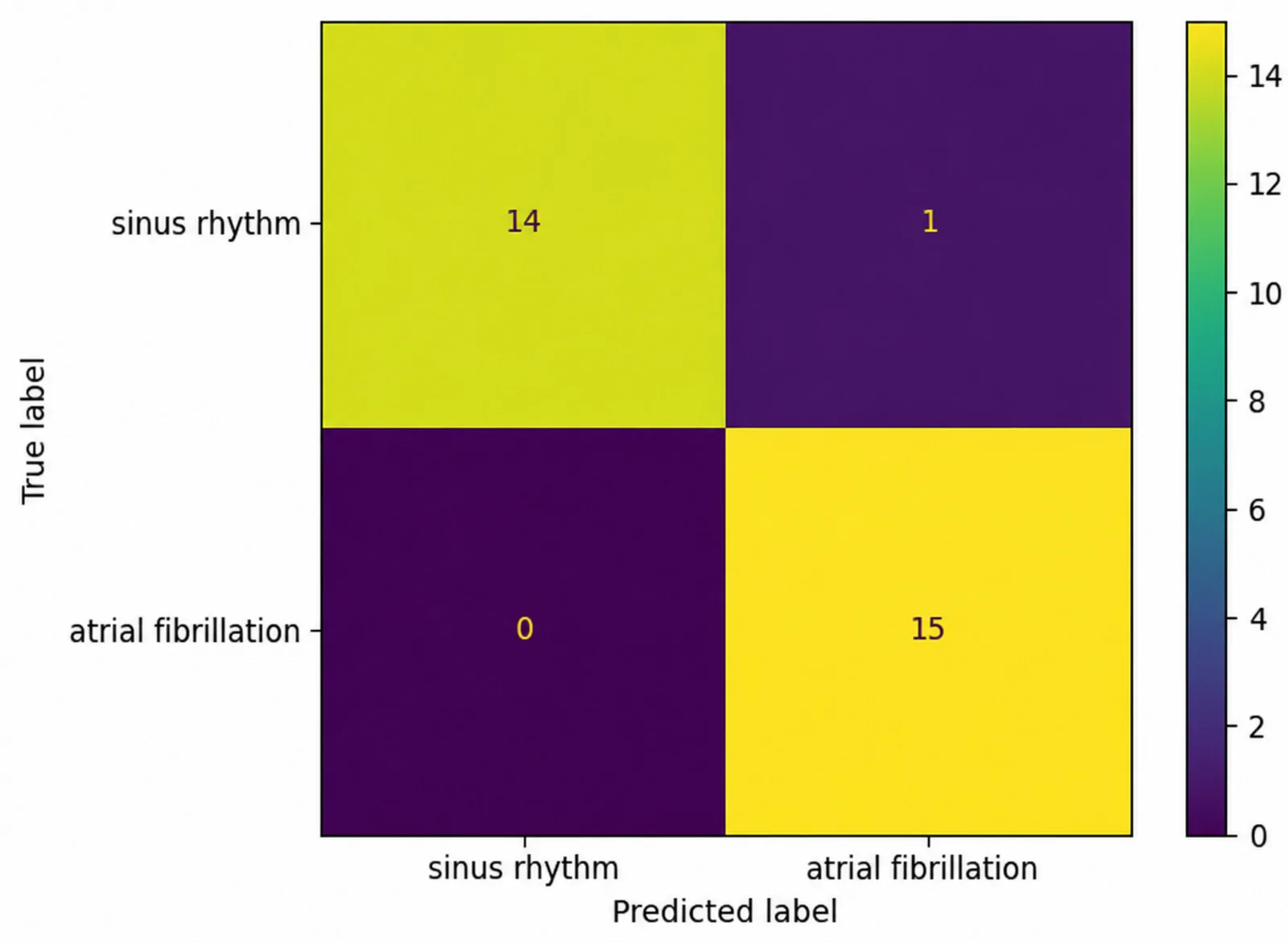
Evaluation of the predictive performance of the model for detecting new-onset atrial fibrillation.

The predictive model was evaluated on an independent test dataset comprising 30 patients (20% of the total cohort of 150 participants). The confusion matrix demonstrated excellent discriminative performance. Among the 15 patients with sinus rhythm, 14 were correctly classified, while only one patient was incorrectly predicted as having atrial fibrillation. Conversely, all 15 patients with atrial fibrillation were correctly identified by the model, with no false-negative classifications. The overall classification accuracy reached 97.0%, with 29 of the 30 test cases correctly classified. For the sinus rhythm class, the model achieved a precision of 1.00, a recall of 0.932, and an F1-score of 0.971. For atrial fibrillation, precision was 0.941, recall reached 1.00, and the corresponding F1-score was 0.971. The macro-average and weighted-average values for precision, recall, and F1-score were all 0.971, indicating balanced performance across both outcome categories (Figure 1).

The discriminatory performance of the prediction model was further evaluated using receiver operating characteristic (ROC) curve analysis. The model achieved an area under the ROC curve (AUC) of 0.991, substantially exceeding the reference line corresponding to random classification (AUC = 0.500). This finding indicates a good ability of the model to distinguish between patients who developed atrial fibrillation and those who remained in sinus rhythm.

Visual inspection of the ROC curve demonstrated that the model consistently maintained high sensitivity across a broad range of specificity thresholds, with the curve closely approaching the upper-left corner of the graph. Such a pattern is characteristic of highly accurate classification models and reflects a very low rate of misclassification.

**Figure 2:**
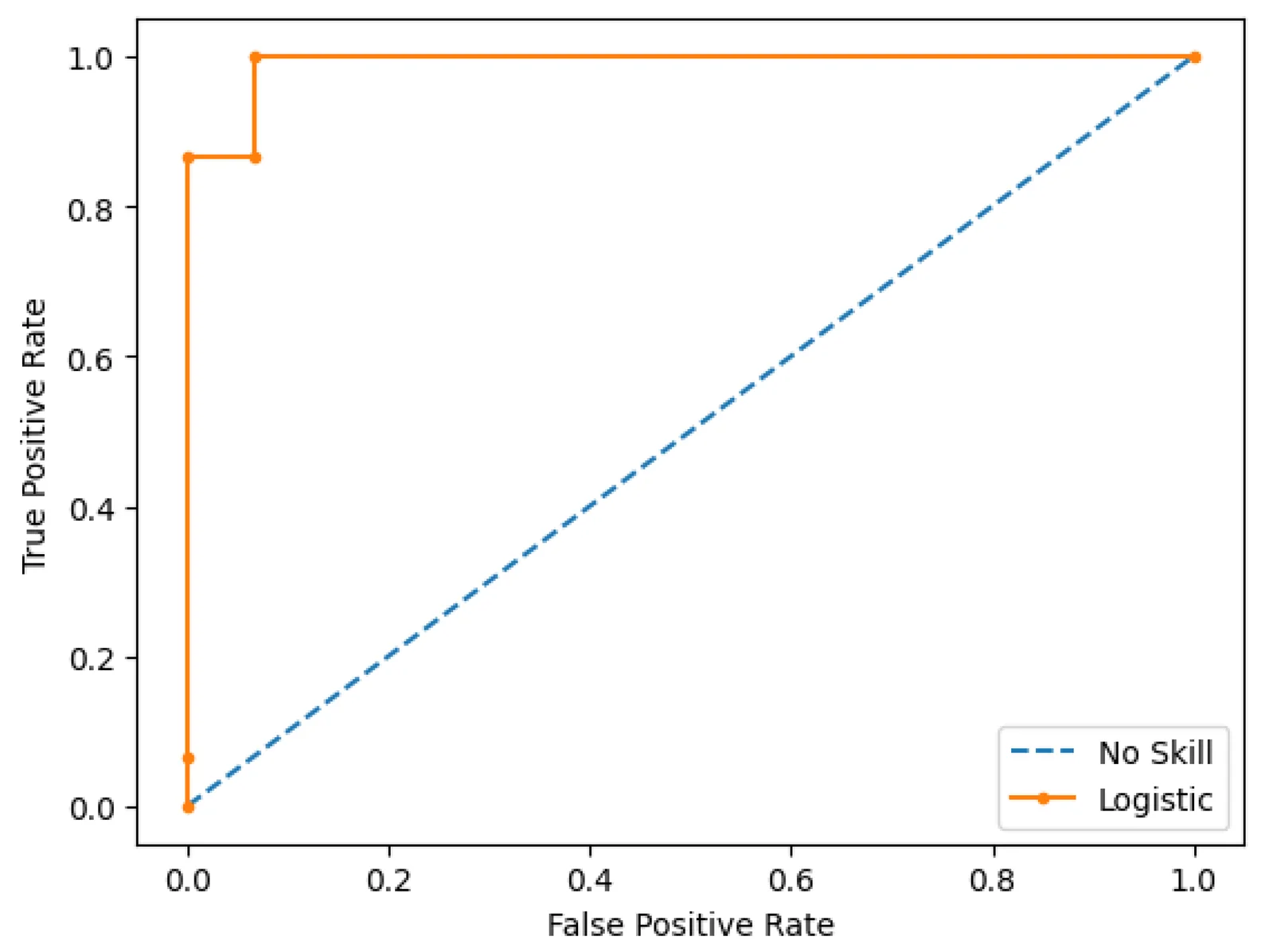
Predictive accuracy of the machine learning model for new-onset atrial fibrillation.

The ROC analysis is consistent with the results obtained from the confusion matrix and classification report, which showed an overall accuracy of 97.0% and only one incorrectly classified patient in the test dataset (Figure 2).

The performance of the prediction model was additionally assessed using a precision–recall (PR) curve. The model achieved an F1-score of 0.968 and an AUC of 0.991, indicating excellent predictive performance. The PR curve remained markedly above the no-skill reference line throughout almost the entire range of recall values, demonstrating a consistently high precision in identifying patients with atrial fibrillation.

Notably, precision remained close to 1.00 across a wide spectrum of recall values, suggesting that the majority of patients classified as having atrial fibrillation were correctly identified. This finding highlights the model’s ability to maintain a favorable balance between sensitivity and positive predictive value, minimizing both false-negative and false-positive classifications.

**Figure 3:**
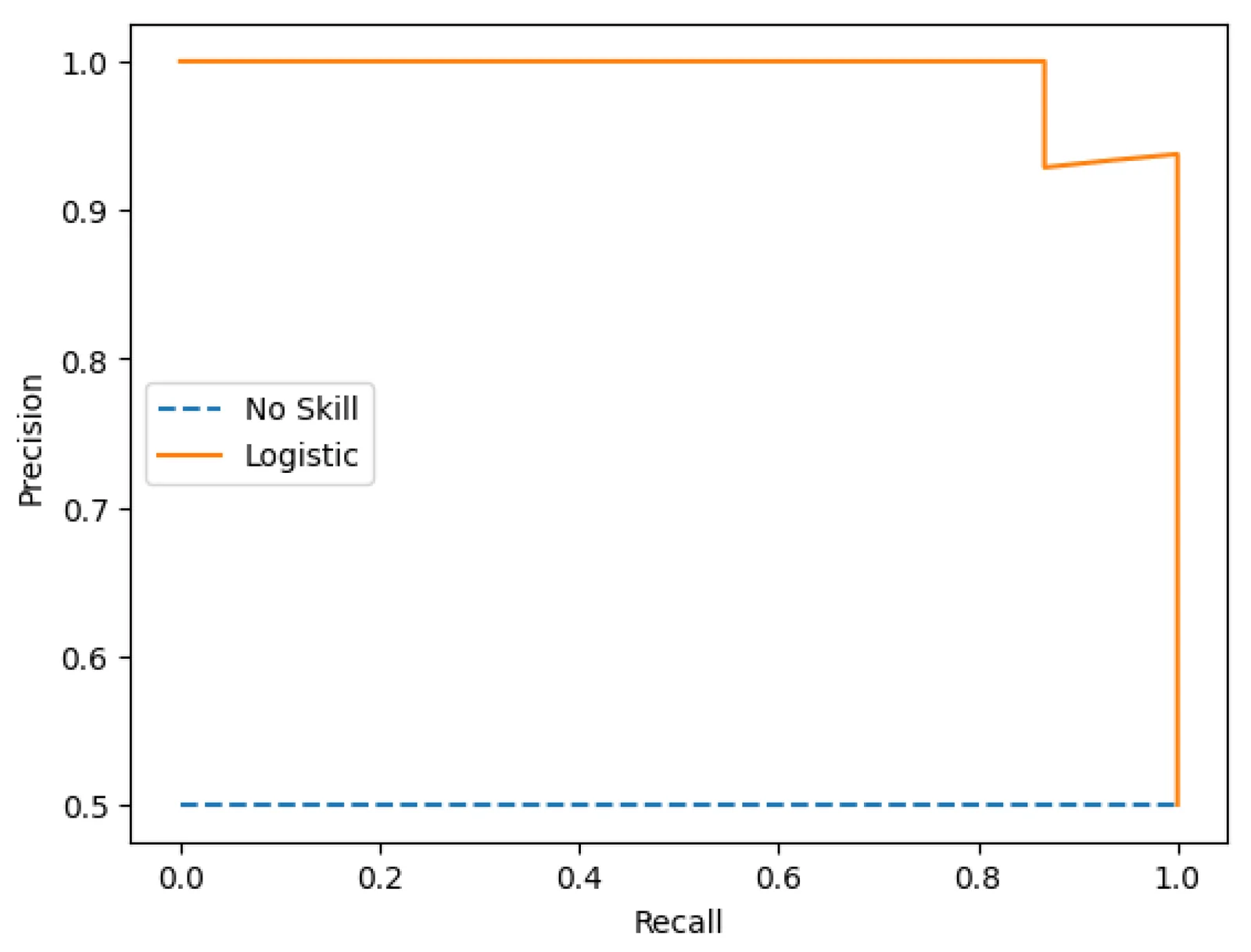
Precision–recall curve for prediction of new-onset atrial fibrillation.

The results of the precision–recall analysis are consistent with those obtained from the ROC curve and confusion matrix, which demonstrated an overall accuracy of 97.0%, an F1-score of 0.971, and only one misclassified patient in the test dataset (Figure 3).

**Figure 4:**
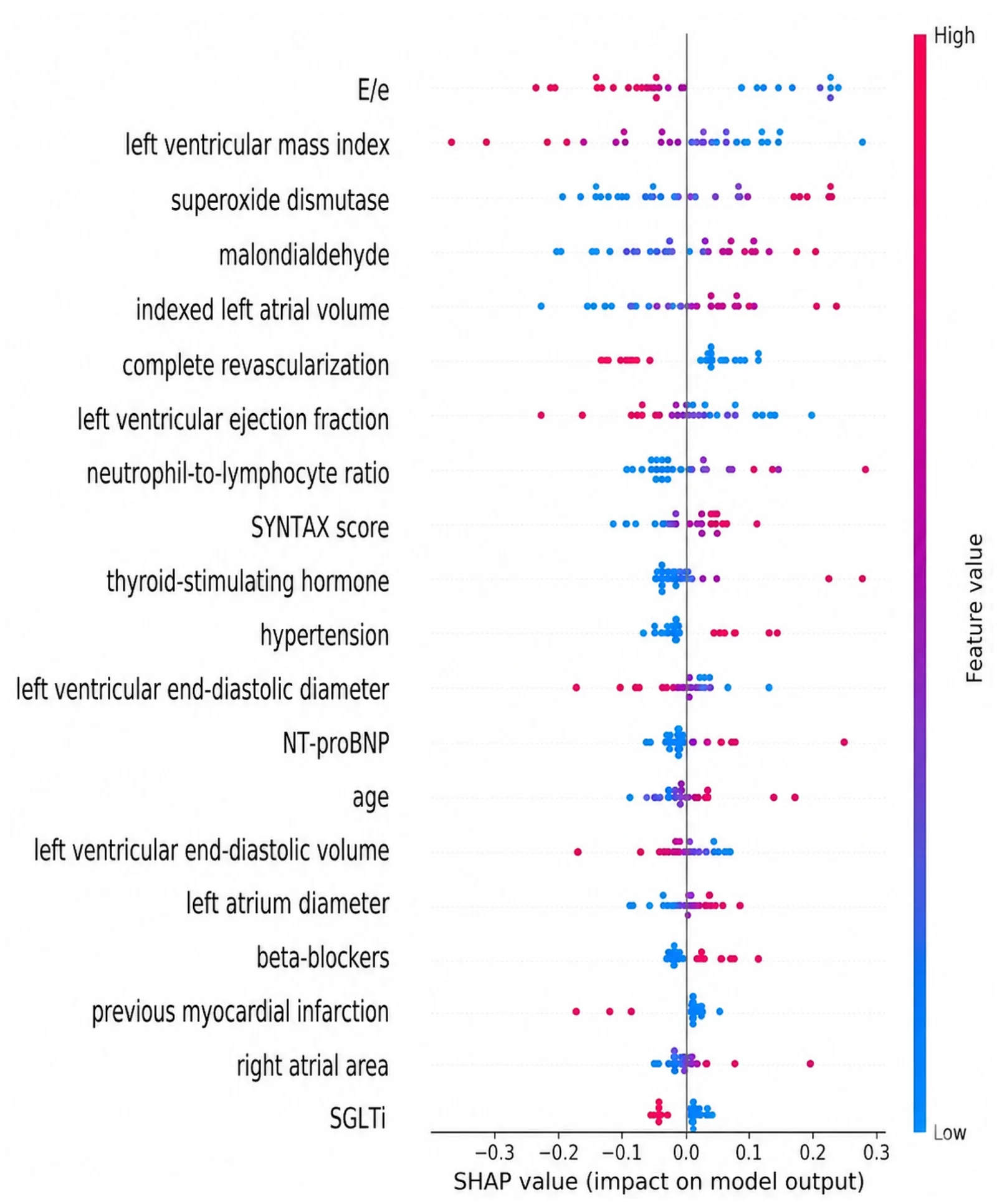
SHAP summary plot of feature importance for predicting new-onset atrial fibrillation.

Figure 4 shows the SHAP summary plot, providing an overview of the relative importance of the predictors and their influence on the model output for new-onset atrial fibrillation. SHAP analysis was employed to enhance model interpretability by quantifying the contribution of each predictor to the final model output. The variables are ordered according to their mean absolute SHAP value, reflecting their overall importance in the prediction process. Each point represents an individual observation, while the position along the x-axis indicates the SHAP value, corresponding to the magnitude and direction of the variable’s effect on the predicted outcome. Positive SHAP values shift the prediction toward a higher probability of the outcome, whereas negative values decrease the predicted probability. The color gradient denotes the actual feature value, with blue representing lower values and red representing higher values.

The analysis revealed that the E/e’ ratio was the most influential predictor in the model. Higher E/e’ values were predominantly associated with positive SHAP values, indicating that elevated left ventricular filling pressures contributed substantially to an increased predicted risk. The second most important variable was left ventricular mass index, which also demonstrated a marked impact on model predictions, with lower values tending to be associated with negative SHAP values and higher values generally shifting predictions toward increased risk. From other echocardiographic parameters with an impact on the prediction model was the left atrial volume indexed to body surface area, with larger values generally contributing to higher model outputs. Left ventricular ejection fraction was another determinant, where lower ejection fraction values were associated with increased risk, while preserved systolic function tended to reduce the predicted probability of the adverse outcome. In addition, ventricular dimensions, represented by left ventricle end-diastolic diameter and left ventricle end-diastolic volume, contributed to model performance, although with lower overall importance compared with the leading variables.

Among the biochemical markers, superoxide dismutase and malondialdehyde emerged as major contributors. Elevated malondialdehyde values, reflecting increased oxidative stress and lipid peroxidation, were associated with positive SHAP values and therefore a higher predicted risk. Conversely, higher superoxide dismutase levels tended to be associated with lower SHAP values, suggesting a protective effect of greater antioxidant capacity. Higher levels of NT-proBNP was associated with increased predicted risk. Likewise, an elevated neutrophil-to-lymphocyte ratio shifted predictions toward higher risk values.

Clinical characteristics also influenced model predictions. Complete revascularization was identified as one of the strongest protective factors, as patients who underwent complete revascularization generally exhibited negative SHAP values, indicating a reduction in predicted risk. Similarly, the presence of beta-blocker therapy and SGLT2 inhibitor treatment tended to be associated with lower model outputs, supporting their beneficial role. Traditional cardiovascular risk factors retained predictive relevance but exerted a smaller effect than the leading variables. Age, hypertension, and a history of previous myocardial infarction contributed to the model output, although their impact was less pronounced. The SYNTAX score, reflecting the complexity of coronary artery disease, also influenced predictions, with higher scores generally associated with increased risk.

To further investigate the relative contribution of individual predictors to the model’s performance, a permutation feature importance analysis was conducted. This method quantifies the importance of each variable by measuring the decrease in predictive accuracy following random permutation of its values, while all other features remain unchanged. Variables associated with larger decreases in model performance are considered more influential for prediction.

The permutation importance analysis identified that left ventricular mass index was one of the most influential predictors, exhibiting the largest median decrease in model accuracy after permutation. This finding indicates that the model relies heavily on this variable for generating accurate predictions. Other highly influential predictors included the E/e’ ratio, left ventricular ejection fraction, and thyroid-stimulating hormone levels, all of which demonstrated substantial reductions in predictive performance when their information was disrupted.

Among the biochemical parameters, markers of oxidative stress, namely superoxide dismutase and malondialdehyde, ranked among the most important variables, suggesting a significant contribution of oxidative stress-related mechanisms to the predictive process. Structural and functional echocardiographic parameters, including left atrial volume indexed to body surface area and left ventricular end-diastolic diameter, also showed meaningful importance scores, highlighting the relevance of ventricular remodeling and cardiac function.

**Figure 5:**
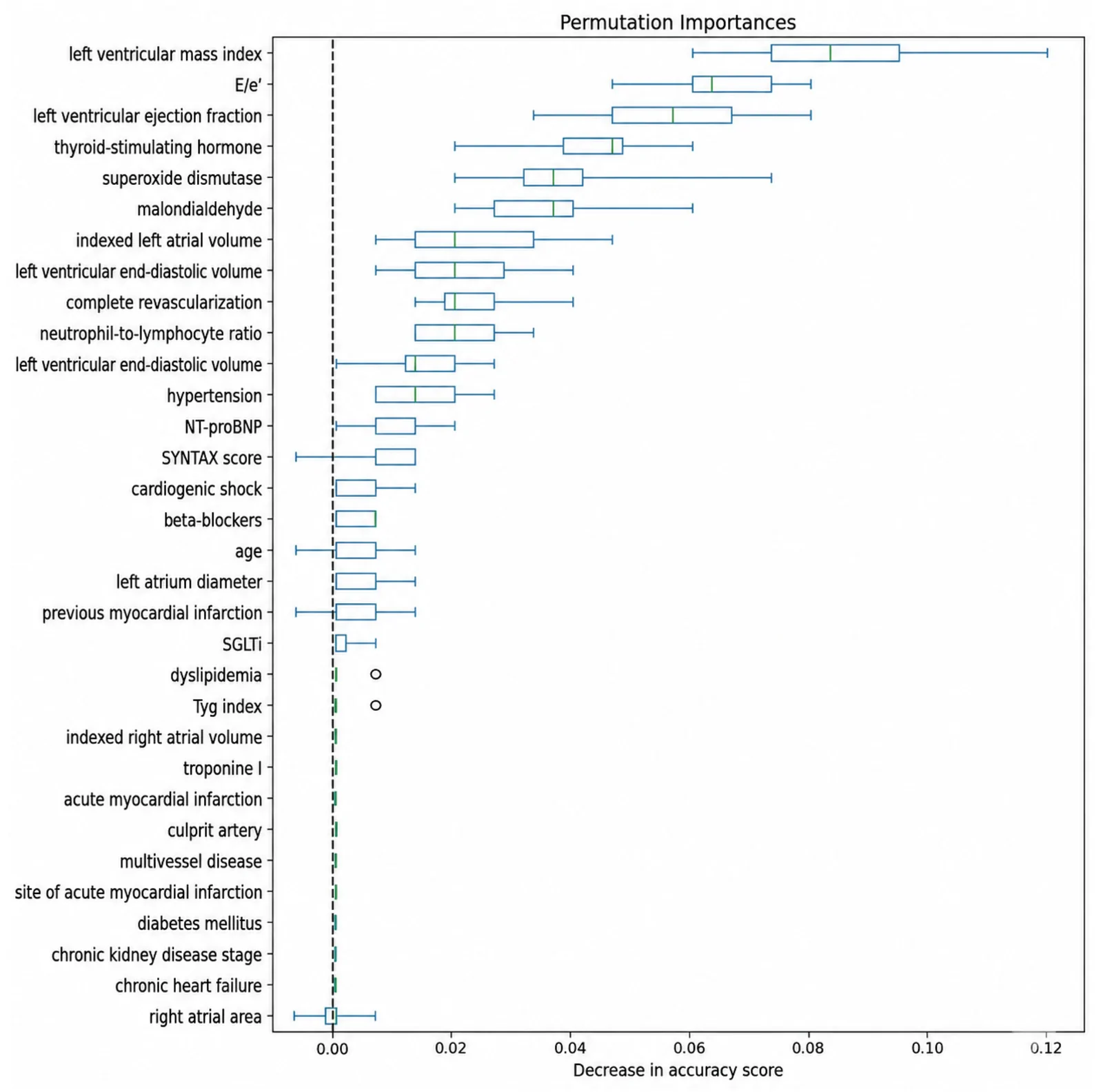
Variable importance for new-onset atrial fibrillation prediction.

Clinical characteristics such as complete revascularization, neutrophil-to-lymphocyte ratio, left ventricular end-diastolic volume, hypertension, NT-proBNP, and SYNTAX score demonstrated moderate predictive contributions. In contrast, variables including troponin I, culprit vessel, infarct localization, multivessel disease, Killip class, and several other conventional clinical parameters displayed near-zero importance values, indicating that their inclusion provided little additional predictive information beyond that already captured by the most influential predictors. The relatively narrow interquartile ranges observed for the highest-ranking variables suggest good stability and reproducibility of the importance estimates across repeated permutations, supporting the robustness of the identified predictor hierarchy (Figure 5).

**Figure 6:**
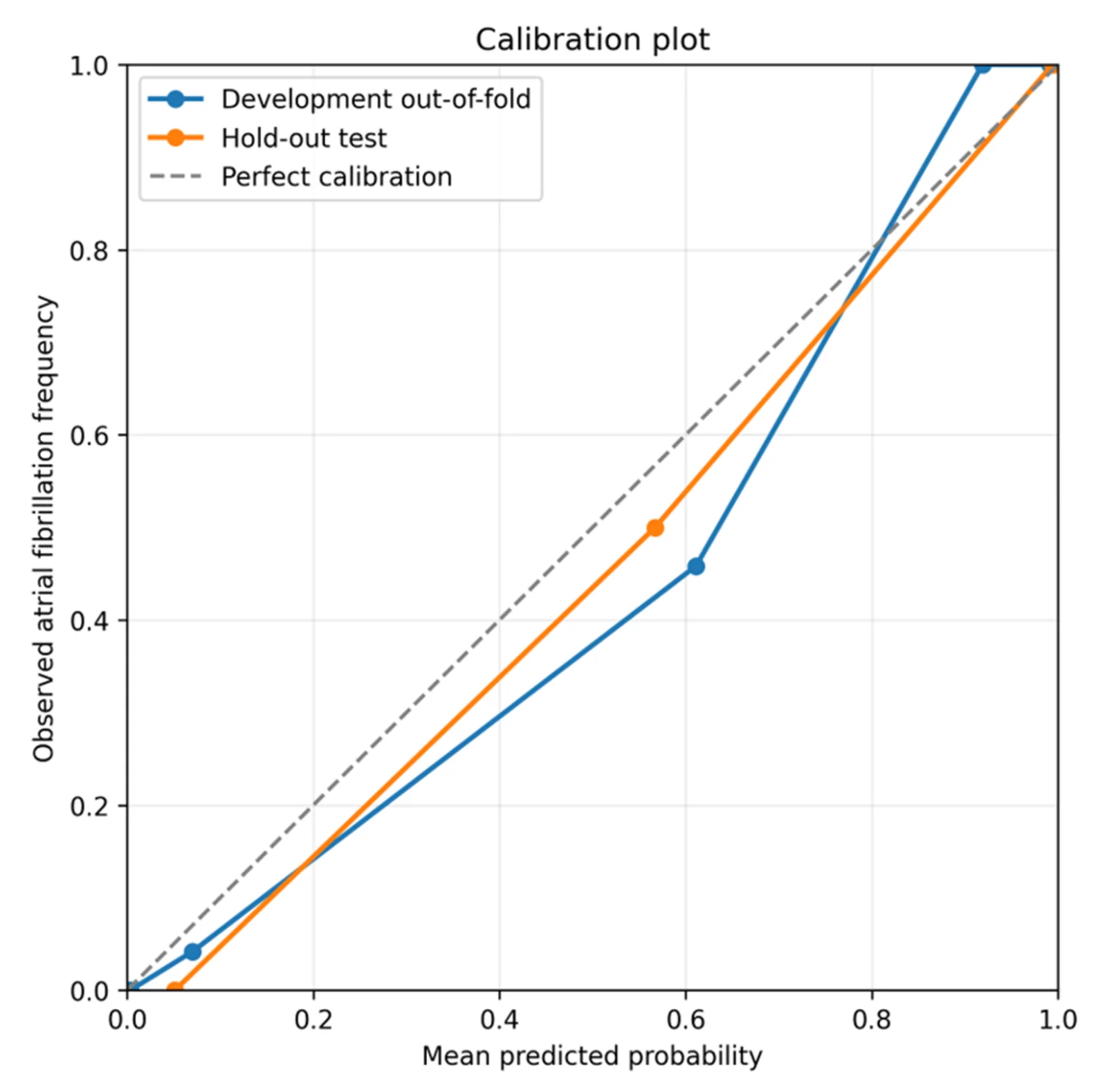
Calibration plot for the final logistic-regression model.

We have also performed a dedicated calibration analysis. The revised notebook included a calibration-analysis step after the ROC and precision–recall evaluations. This analysis calculated the Brier score, calibration intercept, calibration slope, and calibration plot for the final logistic-regression model. Calibration performance was assessed both on the untouched hold-out test set and through repeated out-of-fold predictions within the development cohort, providing a more robust evaluation (Figure 6).

The dashed line represents perfect calibration; the blue curve shows repeated out-of-fold predictions in the development set; and the orange curve shows the independent hold-out test set.

The calibration curves broadly follow the diagonal reference line, which supports reasonable agreement between predicted probabilities and observed atrial fibrillation frequency in this internally validated dataset. The Brier scores were low in both analyses (0.045 for repeated development out-of-fold predictions and 0.036 for the hold-out test set), indicating small average squared prediction error.

However, the calibration intercepts were negative (−1.045 in development out-of-fold assessment and −0.959 in the hold-out test set), suggesting a tendency toward average risk overestimation. The calibration slopes were above one (2.054 and 2.230), suggesting that predicted probabilities may be somewhat conservative, with lower-risk patients not predicted low enough and higher-risk patients not predicted high enough. These estimates must be interpreted cautiously because the hold-out test set included only 30 patients and the full cohort was artificially balanced 1:1, which does not reflect real-world post-AMI atrial fibrillation incidence. A calibration metric summary is presented in Table 3.

## 4. Discussion

The present study developed and validated an explainable artificial intelligence model for predicting new-onset atrial fibrillation in patients with acute myocardial infarction. Our model demonstrated a good predictive performance, with an overall accuracy of 97.0%, an F1-score of 0.971, and an AUC of 0.991. These results compare favorably with previously published prediction models for new-onset atrial fibrillation after AMI, which generally reported AUC values ranging from 0.75 to 0.85 [16,17]. Wu et al. reported an AUC of approximately 0.78 in a model incorporating age, inflammatory markers, and metabolic indices [18], while Zhang et al. developed a nomogram-based model with moderate discriminative ability [9]. Similarly, Wang et al. demonstrated the predictive value of electrocardiographic parameters such as P-wave amplitude in lead V1, although the performance remained lower than that observed in the present study [19].

In this study, we observed the dominant contributions of echocardiographic parameters. According to SHAP analysis, the E/e’ ratio emerged as the strongest predictor of new-onset atrial fibrillation. This observation is biologically plausible because elevated E/e’ reflects increased left ventricular filling pressure, a recognized mechanism promoting left atrial remodeling and atrial fibrillation development [20]. Previous studies have consistently demonstrated that diastolic dysfunction is strongly associated with AF occurrence in patients with cardiovascular disease and following myocardial infarction [21].

Left ventricular mass index was one of the most influential predictors in the permutation importance analysis and one of the leading predictors in the SHAP model. Increased left ventricular mass index reflects chronic myocardial remodeling and hypertrophy, both of which are associated with myocardial fibrosis and atrial structural changes that facilitate AF development. Similar associations between left ventricle hypertrophy and incident AF have been reported in several contemporary studies [22].

Another major echocardiographic determinant was indexed left atrial volume. Left atrial enlargement is considered one of the strongest markers of atrial remodeling and has consistently been associated with both incident and recurrent AF [23]. The importance of this variable in our model further supports the hypothesis that structural atrial abnormalities contribute substantially to the development of new-onset AF after AMI.

Reduced left ventricular ejection fraction also significantly influenced model predictions. Previous investigations have shown that impaired systolic function promotes neurohormonal activation, myocardial fibrosis, and atrial pressure overload, thereby increasing susceptibility to atrial arrhythmias [24]. Our findings are consistent with these observations and reinforce the role of ventricular dysfunction in post-infarction arrhythmogenesis.

An interesting finding of the present study is the prominent role of oxidative stress biomarkers. Malondialdehyde was associated with increased predicted risk, whereas superoxide dismutase exhibited a protective effect. Growing evidence indicates that oxidative stress contributes directly to atrial electrical and structural remodeling through inflammation, mitochondrial dysfunction, calcium-handling abnormalities, and fibrosis [25]. Korantzopoulos et al. and Sagris et al. demonstrated that oxidative stress markers are significantly elevated in patients with atrial fibrillation and may contribute to disease progression [26]. Our results suggest that oxidative stress may also play an important role in new-onset AF development after AMI.

Inflammation appears to represent another important mechanism. In the current model, a higher neutrophil-to-lymphocyte ratio was associated with increased AF risk. This finding is supported by previous studies demonstrating that inflammatory activation contributes to atrial fibrosis and electrical instability [27]. An elevated neutrophil-to-lymphocyte ratio has previously been identified as an independent predictor of new-onset AF in AMI populations.

Among biochemical variables, NT-proBNP was associated with increased risk of new-onset AF. Elevated natriuretic peptide levels reflect myocardial wall stress and have repeatedly been linked to both incident AF and adverse cardiovascular outcomes. The inclusion of NT-proBNP among the most important predictors supports previous evidence highlighting its value for risk stratification. Clinical and therapeutic variables also demonstrated substantial influence. Complete revascularization emerged as one of the strongest protective factors in the model. Previous studies have demonstrated that successful revascularization reduces residual ischemia, adverse remodeling, and recurrent cardiovascular events [28]. Our findings suggest that complete revascularization may additionally reduce the risk of developing new-onset AF. Similarly, beta-blocker therapy was associated with lower predicted risk. This finding is consistent with current evidence supporting beta-blockers as cornerstone therapies for reducing post-infarction arrhythmias through sympathetic modulation [29]. SGLT2 inhibitor treatment also appeared protective, which is in line with recent studies suggesting antiarrhythmic and cardioprotective effects mediated through reductions in inflammation, oxidative stress, and myocardial remodeling [30].

The present study provides the application of explainable artificial intelligence techniques. The use of SHAP analysis enabled identification of the variables driving model predictions and addressed one of the principal limitations of machine learning approaches, namely the lack of interpretability. Similar explainable artificial intelligence approaches have recently been employed in cardiovascular medicine for predicting mortality after myocardial infarction, recurrent atrial fibrillation, and cancer therapy-related cardiac dysfunction [31].

## 5. Conclusions

The development of this machine learning model aimed to improve the identification of patients at increased risk by integrating a wide range of clinical, echocardiographic, angiographic, and biochemical parameters. By simultaneously analyzing multiple variables and their interactions, the model provides a more comprehensive assessment than traditional approaches based on a limited number of predictors. The interpretability analyses showed that markers of cardiac structure and function, oxidative stress, and treatment-related variables played a major role in determining the predicted risk. These findings are consistent with current evidence regarding the multifactorial mechanisms involved in cardiovascular disease progression and support the biological plausibility of the model. An important advantage of the proposed model is its ability to identify patients who may be at higher risk despite apparently similar clinical characteristics. This could facilitate earlier recognition of vulnerable individuals, closer monitoring, and a more personalized therapeutic approach. In addition, the results highlight the potential value of combining conventional cardiovascular risk markers with newer biomarkers and advanced imaging parameters to improve risk prediction.

Overall, our findings suggest that machine learning techniques may represent a useful complementary tool for risk stratification in clinical practice. Although further validation in larger external cohorts is necessary, the present model provides a promising framework for supporting clinical decision-making and improving individualized patient management.

## 6. Limitations

This study has several limitations that should be considered when interpreting the findings. Although the study was prospective, it was conducted at a single tertiary cardiology center and included a relatively small cohort of 150 patients. The limited sample size increases the risk of model overfitting and reduces the precision of performance estimates, particularly given the large number of candidate predictors evaluated. To mitigate this risk, candidate machine learning algorithms were compared using repeated stratified cross-validation within the development cohort before final evaluation on an independent internal test set. Nevertheless, the test set comprised only 30 patients (15 with and 15 without new-onset atrial fibrillation), limiting the number of outcome events and rendering performance metrics, including accuracy, ROC-AUC, and precision–recall AUC, potentially unstable and optimistic. Accordingly, the high discriminatory performance observed should be interpreted with caution.

The model was both developed and internally tested using data from the same institutional cohort, and external validation in geographically and clinically distinct populations was not performed. Furthermore, the study employed a balanced case–control-like design with equal numbers of patients with and without new-onset atrial fibrillation. While this approach facilitates algorithm development and comparison between groups, it does not reflect the true incidence of atrial fibrillation after acute myocardial infarction and may affect model calibration, predictive values, and clinical applicability in routine practice.

Several potentially informative predictors were not systematically assessed, including cardiac magnetic resonance imaging parameters, atrial strain measurements, genetic markers, and longitudinal biomarker trajectories. In addition, the proposed machine learning model was not directly compared with established clinical risk scores or conventional statistical prediction models. Future studies should determine whether machine learning provides incremental predictive value beyond existing risk stratification approaches.

Finally, SHAP and permutation feature importance analyses were used to improve model interpretability rather than to establish association relationships. Differences between feature rankings obtained with these methods were expected because they evaluate predictor importance using different methodological principles and because several clinical and echocardiographic variables are correlated. Although feature-ranking stability was assessed through repeated resampling, the relative importance of individual predictors varied across folds. Therefore, the identified predictors should be regarded as candidate robust markers requiring confirmation in larger external cohorts rather than definitive mechanistic determinants. A limitation of this study is that patients with STEMI and NSTEMI were analyzed as a single acute myocardial infarction cohort without separate subgroup analyses. Therefore, potential differences between these populations were not specifically evaluated.

Overall, these findings should be considered preliminary. Large prospective multicenter studies with external validation, calibration assessment, decision-curve analysis, and evaluation across clinically relevant risk thresholds are required before implementation of the model in routine clinical practice can be recommended.

## Figures and Tables

**Table 1:** Baseline demographic and clinical characteristics.

	Total Cohort, *n* = 150 (%)	CI 95%
Previous stroke	15 (10.0%)	5.2%, 15%
Peripheral artery disease	7 (4.7%)	1.3%, 8.0%
Smoking	49 (32.7%)	25%, 40%
Alcohol consumption	43 (28.7%)	21%, 36%
Arterial hypertension	102 (68.0%)	61%, 75%
Hypertension grade		
Absence of hypertension	48 (32.0%)	25%, 39%
Grade I	3 (2.0%)	0.00%, 4.2%
Grade II	52 (34.7%)	27%, 42%
Grade III	47 (31.3%)	24%, 39%
Previous myocardial infarction	15 (10.0%)	5.2%, 15%
Chronic heart failure	42 (28.0%)	21%, 35%
Type 2 diabetes mellitus	37 (24.7%)	18%, 32%
Anemia	28 (18.7%)	12%, 25%
Dyslipidemia	124 (82.7%)	77%, 89%
Metabolic syndrome	36 (24.0%)	17%, 31%
Chronic kidney disease	21 (14.0%)	8.4%, 20%
Chronic obstructive pulmonary disease	8 (5.3%)	1.7%, 8.9%
Thyroid disease	7 (4.7%)	1.3%, 8.0%
Malignant neoplastic disease	3 (2.0%)	0.0%, 4.2%

**Table 2:** General characteristics and comorbidities according to study group.

	Group I (Newly Onset AF), *n* = 75 ^1^, *n* (%)	95% CI	Group II (Sinus Rhythm), *n* = 75, *n* (%)	95% CI	Test Statistic ^2^	*p*-Value ^2^
Previous stroke	9 (12.0%)	4.6%, 19%	6 (8.0%)	1.9%, 14%	0.67	0.6
Peripheral artery disease	7 (9.3%)	2.7%, 16%	0 (0.0%)	0.00%	7.3	0.014
Smoking	30 (40.0%)	29%, 51%	19 (25.3%)	15%, 35%	3.7	0.082
Hypertension grade					12	0.004
Absence of hypertension	27 (36.0%)	25%, 47%	21 (28.0%)	18%, 38%		
Grade I	2 (2.7%)	0.00%, 6.3%	1 (1.3%)	0.00%, 3.9%		
Grade II	16 (21.3%)	12%, 31%	36 (48.0%)	37%, 59%		
Grade III	30 (40.0%)	29%, 51%	17 (22.7%)	13%, 32%		
Previous myocardial infarction	4 (5.3%)	0.25%, 10%	11 (14.7%)	6.7%, 23%	3.6	0.10
Chronic heart failure	24 (32.0%)	21%, 43%	18 (24.0%)	14%, 34%	1.2	0.4
Type 2 diabetes mellitus	22 (29.3%)	19%, 40%	15 (20.0%)	11%, 29%	1.8	0.3
Anemia	24 (32.0%)	21%, 43%	4 (5.3%)	0.25%, 10%	18	<0.001
Dyslipidemia	53 (70.7%)	60%, 81%	71 (94.7%)	90%, 100%	15	<0.001
Metabolic syndrome	19 (25.3%)	15%, 35%	17 (22.7%)	13%, 32%	0.15	0.8
Chronic kidney disease	13 (17.3%)	8.8%, 26%	8 (10.7%)	3.7%, 18%	1.4	0.3
Chronic obstructive pulmonary disease	4 (5.3%)	0.25%, 10%	4 (5.3%)	0.25%, 10%	0.0	>0.9
Thyroid disease	5 (6.7%)	1.0%, 12%	2 (2.7%)	0.00%, 6.3%	1.3	0.4
Malignant neoplastic disease	0 (0.0%)	0.00%	3 (4.0%)	0.00%, 8.4%	3.1	0.2

Note: ^1^ *n* (%), mean (SD), median (IQR), minimum, maximum; ^2^ Pearson’s χ^2^ test with simulated *p*-value; Wilcoxon rank-sum test.

**Table 3:** Calibration metric summary.

Dataset	*n*	Events	Brier Score	Calibration Intercept	Calibration Slope
Development out-of-fold	120	60	0.045	−1.045	2.054
Hold-out test	30	15	0.036	−0.959	2.230

## Data Availability

The original contributions presented in this study are included in the article. Further inquiries can be directed to the corresponding author.

## Abbreviations

AF	atrial fibrillation
AMI	acute myocardial infarction
AUC	area under the curve
ROC	receiver operating characteristic curve
NLR	neutrophil-to-lymphocyte ratio
NT-proBNP	N-terminal pro–B-type natriuretic peptide
SYNTAX	synergy between PCI with taxus and cardiac surgery score
SHAP	SHapley Additive Explanations
